# Sensory and Physical Properties of Fibrous Meat Analogs Made from Faba Bean, Pea, and Oat Using High-Moisture Extrusion

**DOI:** 10.3390/foods13101444

**Published:** 2024-05-08

**Authors:** Antti Knaapila, Katja Kantanen, Jose Martin Ramos-Diaz, Vieno Piironen, Mari Sandell, Kirsi Jouppila

**Affiliations:** 1 Department of Food and Nutrition, University of Helsinki, P.O. Box 66, Agnes Sjöbergin katu 2, FI-00014 Helsinki, Finland; katja.kantanen@helsinki.fi (K.K.); martin.ramosdiaz@luke.fi (J.M.R.-D.); vieno.piironen@helsinki.fi (V.P.); mari.sandell@helsinki.fi (M.S.); kirsi.jouppila@helsinki.fi (K.J.); 2 Natural Resources Institute Finland (Luke), Humppilantie 7, FI-31600 Jokioinen, Finland

**Keywords:** CATA, consumers, hedonic value, meat alternative, plant-based protein, sensory evaluation, texture profile analysis, *Vicia faba*

## Abstract

Faba bean is a promising source of ingredients for the production of meat analogs. However, sensory properties of faba bean, especially the bitter taste of the protein concentrate, restrict its use. Our aim was to assess the feasibility of two types of faba bean ingredients—flour (from germinated, gently heat-treated beans) and groat (from non-germinated, roasted beans)—in combination with pea protein isolate and oat fiber concentrate for producing meat analogs using high-moisture extrusion. We produced six samples using varying recipes, while maintaining constant process parameters. An untrained panel (55 participants) evaluated the samples for key sensory attributes (check-all-that-apply) and rated their pleasantness. The water absorption capacity and mechanical properties of the samples were assessed using instrumental measurements. The samples were frequently described as ‘beany’ and ‘tasteless’, but very rarely as ‘bitter’. The most frequently cited attributes for mouthfeel varied between the samples containing 30% (‘tough’, ‘gummy’) and 50% (‘crumbly’, ‘floury’) of faba bean flour/groat and were associated with corresponding mechanical properties. On average, the sample containing a blend of faba bean groat and pea protein isolate (50% each) appeared to be the most pleasant. Our results suggest that faba bean groat with pea protein isolate enables the production of fibrous meat analogs with acceptable taste and texture, without the bitter off-taste.

## 1. Introduction

Alternatives for conventional meat products include analogs made from plant-based sources (such as legumes and oilseeds), mycoprotein, algae, insects, and cultured meat [1]. Among the different options, plant-based meat analogs appear to be the easiest for consumers to accept ([2], for reviews, see [3,4]). The relative preference for plant-based options fuels demand for the plant-based meat analogs [5,6].

High-moisture extrusion (HME) technology (50–70% moisture) has become popular in manufacturing structured plant-based meat analogs [7]. Production of fibrous, meat-mimicking texture requires an extruder with a long cooling die and a recipe high enough in protein (>50% of dry ingredients). Thus, protein-rich ingredients, such as plant-derived protein concentrates and isolates, are typically used [8].

Soybean (*Glycine max*), wheat (*Triticum aestivum*), and pea (*Pisum sativum*) have been the most common and intensively studied sources of protein-rich ingredients for plant-based meat analogs [9]. Recently, other high-protein crops such as faba bean (*Vicia faba*) have gained increasing research interest. Faba bean protein concentrate has been successfully used to produce fibrous meat analogs using HME [10,11,12]. However, the sensory quality of ingredients made from faba bean, especially the protein concentrate (i.e., the protein-rich fraction from dry fractionation of the flour), is compromised by their characteristic bitter taste, which persists into the extrudates (meat analogs) [13].

While compounds underlying the bitter taste of faba bean ingredients have not been definitely identified, several candidates have been proposed. Tuccillo et al. [13] linked the presence of free phenolics, vicine, and convicine to the bitterness of faba bean protein concentrate. In addition to vicine and convicine, Karolkowski et al. [14] observed that saponin content (especially soyasaponin βb) was associated with the bitter taste of faba bean. Furthermore, lipid oxidation may not only contribute to the beany (or pea-like) off-flavor [15], but also to the bitter taste through non-volatile oxidized fatty acids, such as epoxy and hydroxy fatty acids, as in the case of oat [16] and pea [17,18]. Thus, inactivation of lipase and lipid oxidizing enzymes of faba bean material by a heat treatment early enough in the manufacturing process may result in less bitter ingredients.

Germination is another treatment of raw materials that could improve the sensory qualities of plant-based protein ingredients [19,20]. Furthermore, germination can reduce the content of raffinose family oligosaccharides in legume-based ingredients, resulting in less gastrointestinal discomfort to consumers [21].

Among the ingredients from faba bean (flour, protein concentrate, protein isolate), flour has been shown to be less bitter than the protein concentrate [13]. However, faba bean flour (containing ~30% protein on a dry basis [22]) alone will not provide enough protein to form a fibrous meat analog in HME. Thus, the faba bean flour should be used together with a high-protein ingredient, such as pea protein isolate (containing ~90% protein on a dry basis [23]).

Hydrocolloids (i.e., substances that can form a gel-like structure when they come into contact with water), such as gellan gum, pectin, and xanthan, may be beneficial for the texture formation and sensory properties of meat analogs [24]. Recently, Wang et al. [25] showed that a hydrocolloid, dextran, produced in situ by *Weissella confusa* A16 or in vitro, significantly reduced the intensity of bitter taste in faba bean protein concentrate (suspension in water) and the corresponding extrudate. *β*-Glucan is a hydrocolloid that may improve the gel properties of a variety of food products (for a review see [26]), and thus, may also be beneficial to the texture of meat analogs [27]. In addition to technological benefits, *β*-glucan could increase the nutritional value of meat analogs, since *β*-glucan is a dietary fiber with potential health benefits, such as a cholesterol lowering effect [28]. Thus, oat fiber concentrate, a fraction of oat flour that is rich in *β*-glucan, is a promising ingredient for meat analogs. 

Most sensory studies on plant-based meat analogs have investigated the samples made from soybean-derived ingredients (for reviews, see [29] and page 2914 in [30]). Little is known about the effects of faba bean flour (from heat-treated non-germinated or germinated beans) and oat fiber concentrate on the sensory and physical properties of meat analogs made using HME.

The aim of the study was to reveal the effects of faba bean ingredients, pea protein isolate, and oat fiber concentrate, when used in different ratios in HME, on the sensory and physical properties, and pleasantness of the produced meat analogs. We hypothesized that using faba bean flour/groat from heat-treated (non-germinated or germinated) beans, along with pea protein isolate and oat fiber concentrate (rich in *β*-glucan), and applying selected process parameters in HME, it would be possible to produce meat analogs that have an acceptable, neutral taste (without bitter off-taste) and a meat-mimicking fibrous texture.

## 2. Materials and Methods

### 2.1. Samples

Six samples of meat analogs were prepared using ingredients and HME, as detailed below. The samples differed only in their formulation. The process parameters were maintained constant. The same samples were used for sensory and instrumental analyses.

#### 2.1.1. Ingredients

Two types of faba bean ingredients were used: groat (crushed beans) from non-germinated heat-treated (roasted) beans (F) and flour from germinated and gently heat-treated beans (GF). The faba bean ingredients were not fractionated. The roasted faba bean groat (F) was purchased from Vihreä Härkä (Littoinen, Finland) and the flour from germinated faba bean (GF) was purchased from Viking Malt Ltd. (Lahti, Finland). Pea protein isolate (PPI) was purchased form Roquette Freres (Lestrem, France) and oat fiber concentrate (OFC) from Fazer Mills (Lahti, Finland).

#### 2.1.2. Preparation

The six extrudates were prepared with various ratios of F, GF, PPI, and OFC, as presented in Table 1. The protein content of the extrudates was calculated based on the protein content of raw materials analyzed by the Kjeldahl method using Kjeltec digestor and analyzer units (FOSS analytical A7S, Hilleröd, Denmark) using a nitrogen-to-protein conversion factor of 5.4. Extrudates were prepared with high-moisture extrusion using a twin-screw extruder (Rheomex PTW24/28p, Polylab System, Rheocord 300p; Thermo Haake, Karlsruhe, Germany) with a long cooling die (flat cooling nozzle FKD75, DIL Deutsches Institut für Lebensmitteltechnik, Quakenbrück, Germany) [27]. All extrudates were prepared using the same extrusion conditions with 400 rpm screw speed, 85 g/min total feed rate, and 55% feed water content. The temperature profile of the barrel from zones 1–7 was 25, 40, 80, 100, 120, 150, and 150 °C, respectively, and the temperature of the long cooling die was 80 °C. The collected samples were placed in polyethylene zip-lock bags and stored in a freezer at −20 °C until further analysis. We considered storing the extrudates as frozen necessary because they were perishable, and it was not practically possible to prepare them all on the same day.

### 2.2. Sensory Evaluation

#### 2.2.1. Procedure

The six samples were evaluated by an untrained panel consisting of 55 volunteer students enrolled in a sensory science course at the University of Helsinki, Department of Food and Nutrition. The participants were adults, with the majority being women. However, we did not collect demographic data from the participants, as our aim was not to study different consumer segments.

The participants evaluated the samples for sensory properties and pleasantness as detailed below. The samples were presented with 3-digit blind codes in randomized orders. The participants were instructed to rinse their mouths with water between the evaluations of different samples. The sensory evaluation took place in evaluation booths within a sensory laboratory that conformed to the ISO 8589 standard [31]. Participant responses were recorded using printed forms (in Finnish) and subsequently transferred into digital format for statistical analysis (see Section 2.4).

We adhered to the ethical principles of sensory research at the Department of Food and Nutrition, University of Helsinki. These principles were reviewed by the Research Ethics Committee in the Humanities and Social and Behavioural Sciences of University of Helsinki (Statement 15/2020). Written informed consent was obtained from each participant prior to their inclusion in the study.

#### 2.2.2. Check-All-That-Apply

The check-all-that-apply (CATA) technique was applied for rapid sensory profiling of the samples. In this technique, participants are instructed to select all sensory attributes from a provided list that they perceived in the sample being evaluated. For our study, we provided the participants with a list of 14 attributes intended to encompass taste, flavor, and texture aspects of interest (Table 2).

#### 2.2.3. Pleasantness

A 9-point hedonic scale was used to assess the hedonic value (pleasantness) of the samples across five aspects: appearance, odor, texture (evaluated by hand), mouthfeel, and overall pleasantness. The scale was presented horizontally, with hedonic value increasing from left (1) to right (9) on the scale. The classical 9-point hedonic scale is labeled with terms derived from ‘liking’ and ‘disliking’ (for details, see Lawless and Heymann [32], p. 326). However, in our study, the score sheet was provided to the participants in Finnish and because there is no direct equivalent for the term ‘dislike’ in Finnish, we used the Finnish equivalents for ‘pleasant’ and ‘unpleasant’ instead (Table 3). Consequently, throughout the present study, we referred to pleasantness (rather than liking) as the measure of hedonic value.

### 2.3. Physical Measurements

#### 2.3.1. Mechanical Properties

Texture profile analysis (TPA) and cutting strength tests were performed with a texture analyzer (Stable Micro Systems, Godalming, Surrey, England) according to the method by Ramos Diaz et al. [27] with some modifications. The cut sample pieces were placed in a plastic cup with a cover and thawed in an oven for 45 min at 40 °C. The samples were allowed to reach the room temperature before the analysis. TPA was conducted to measure the hardness, gumminess, springiness, and chewiness of the extrudates while cutting strength analysis was performed to measure the longitudinal and perpendicular cutting strengths. Five replicates were measured for TPA and cutting strengths.

#### 2.3.2. Water Absorption Capacity

Water absorption capacity (WAC) was analyzed according to the method by Tuccillo et al. [13]. The extrudates were cut into pieces (width 20 mm, length 30 mm, and height 10 mm), freeze-dried for 3 days, and finally rehydrated in 40 mL Milli-Q water and incubated at 50 °C for 16 h. Three replicates were measured, and the water absorption capacity was calculated as grams of water retained by a gram of freeze-dried extrudate.

### 2.4. Photography

For the photography, the extrudates were cut into 6 cm-long pieces and then carefully opened to show the inner structure. The images were taken with a DSLR body (Nikon 7200, Tokyo, Japan) digital camera with a telephoto lens (18–400 mm, f/3.5–6.3, DI II VC HLD zoom objective, Tamron Co., Ltd., Saitama, Japan) in a light-controlled cabinet using a warm daylight (D50). 

### 2.5. Statistical Analysis

The statistical differences among the samples in pleasantness and mechanical properties of hardness, gumminess, springiness, chewiness, and longitudinal cutting strength were analyzed using one-way analysis of variance (ANOVA) with Tukey’s honestly significant difference as a post hoc test. Perpendicular cutting strength was analyzed using the Kruskal–Wallis test due to non-normal distribution. These analyses were run using IBM SPSS Statistics, version 29 (Armonk, NY, USA). The criterion for statistical significance was set at α = 0.05.

Partial least squares regression analysis (PLSR) was conducted to study the relationships of mechanical properties and WAC with the texture-related CATA attributes of gummy, chewy, crumbly, and floury. The PLSR was performed using SIMCA 17.0 software (Sartorius Stedim Data Analytics AB, Umeå, Sweden).

## 3. Results

### 3.1. Sensory Properties

**Figure 1:**
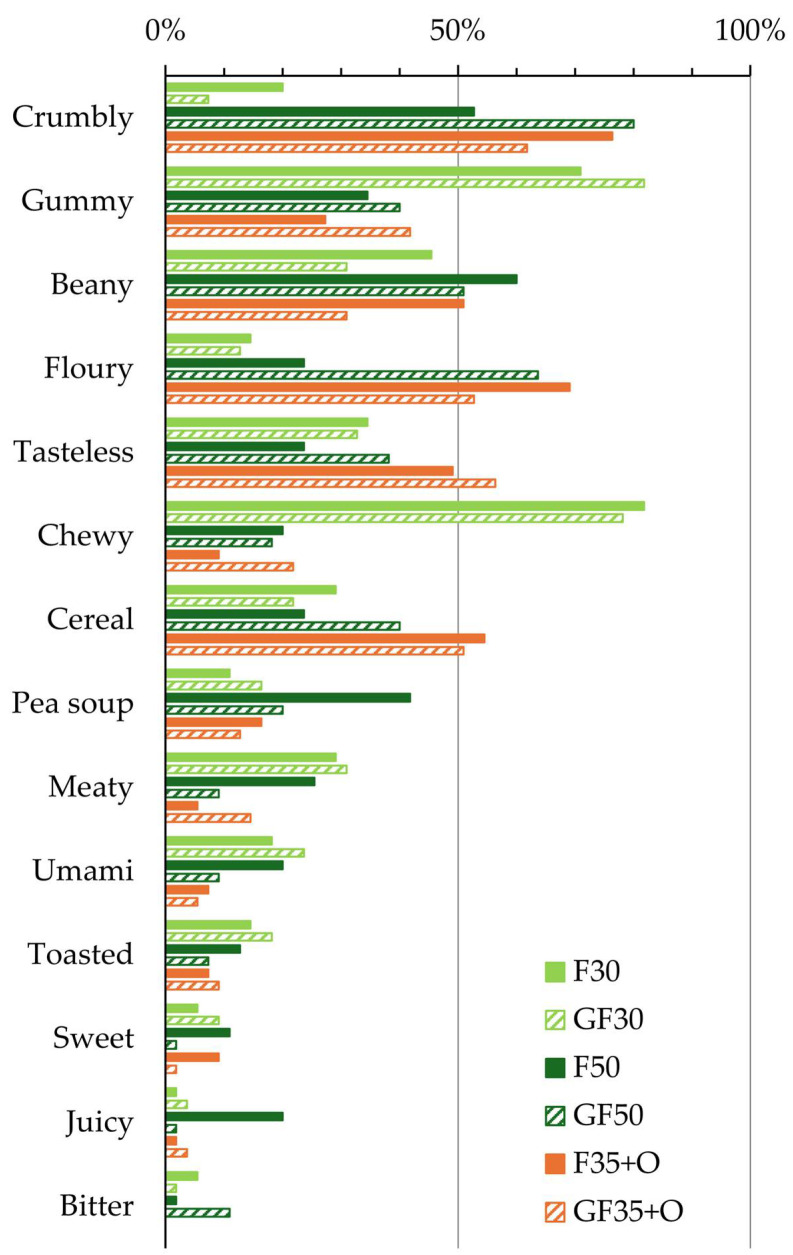
Sensory profiles of the extrudates according to the check-all-that-apply (CATA) task. In CATA, the participants selected all attributes from the provided list (see Table 2) that applied to a sample based on the sensory evaluation. Bars show the percentage out of the 55 participants who selected an attribute to describe a sample (the attributes are sorted in descending order of the average citation frequency). Samples contained faba bean groat (F) or faba bean flour made of germinated beans (GF); number indicates the percentage (30, 35, or 50%). Two of the samples (F35+O and GF35+O) included 30% oat fiber concentrate, and the rest (45–70%) were pea protein isolate (percentages are of dry matter; see Table 1 for details).

The results of the sensory profiling of the extrudate samples using CATA, with the attributes sorted in descending order of the average citation frequency, are shown in Figure 1. The two most frequently cited sensory attributes on average among all samples were ‘crumbly’ and ‘gummy’. However, clear differences among the samples were observed regarding the mouthfeel attributes. The samples containing a low proportion of faba bean groat/flour (and, respectively, a high proportion of pea protein isolate and thus, protein), F30 and GF30, were frequently mentioned as ‘gummy’ and ‘chewy’, but seldom as ‘crumbly’. In contrast, the other samples (F50, F35+O, GF50, GF35+O) were frequently cited as ‘crumbly’, sometimes as ‘gummy’, but seldom as ‘chewy’.

The least frequently cited mouthfeel attribute, on average, was ‘juicy’. Unlike the mouthfeel attributes for which the citation frequency varied a lot among the samples (‘crumbly’, ‘gummy’, and ‘chewy’), none of the samples was frequently described as ‘juicy’. Likewise, ‘meaty’ and ‘umami’ were rarely selected as attributes to describe a sample.

The taste and flavor attributes were rather evenly selected among the samples. The most frequently selected taste/flavor attributes, on average, were ‘beany’ and ‘tasteless’. This suggests that the characteristic beany flavor of faba bean persisted into the meat analogs. Furthermore, the samples including OFC (F35+O, GF35+O) were relatively often mentioned as having a ‘cereal’ note. On the other hand, the samples were very seldom described as ‘bitter’. This implies that bitterness in meat analogs made from faba bean ingredients (which is a common challenge in meat analogs made of faba bean protein concentrate), could be avoided by using faba bean groat/flours from heat-treated, non-germinated or germinated, faba beans.

### 3.2. Pleasantness

Pleasantness of all aspects of the extrudate samples was rated, on average, around the middle of the scale, ranging from slightly unpleasant (3.8) to slightly pleasant (5.8) (Table 4). In all aspects of pleasantness, except for odor, there were significant differences between the samples (ANOVA, *p* < 0.05).

A higher proportion of faba bean groat/flour tended to be associated with a less pleasant appearance. However, our study focused on taste and texture, not appearance, and we did not include attributes related to appearance in the CATA profiling. Thus, we were not able to reveal reasons underlying the differences in pleasantness of appearance.

Pleasantness for texture evaluated by hand and pleasantness of the mouthfeel (i.e., texture evaluated in the mouth) were, in most cases, similar. Among samples made of faba bean groat (F30, F50, F35+O), a higher proportion of the faba bean led to a more pleasant texture and mouthfeel, whereas among the samples made of faba bean flour from germinated beans (GF30, GF50, GF35+O), no difference in pleasantness for texture and mouthfeel was observed. 

The mouthfeel of sample F50 was rated as significantly more pleasant than that of the other samples (5.8 compared to <5). It could be that the textural properties suggested by the CATA (Figure 1) underlaid the pleasantness of the mouthfeel of F50. This sample was rarely described as ‘chewy’ or ‘floury’, and only occasionally as ‘crumbly’ and ‘gummy’, suggesting that these properties may have been at more desirable levels in F50 than in the other samples.

Sample F50 was also rated (at least nominally) as the most pleasant in taste (4.8) and overall (5.1), with a significant difference observed only in comparison with sample GF50, which was rated nominally as the least pleasant in taste (3.8) and overall (3.8) (Table 4). Interestingly, the formulations of these two samples differed only in the type of faba bean ingredient. The results suggest that a more pleasant sensory experience can be provided by a meat analog made using faba bean groat from the non-germinated beans than flour from the germinated beans. However, the difference between the samples was observed only when the proportion of the faba bean ingredient was high (50%).

### 3.3. Mechanical Properties and Water Absorption Capacity

**Figure 2:**
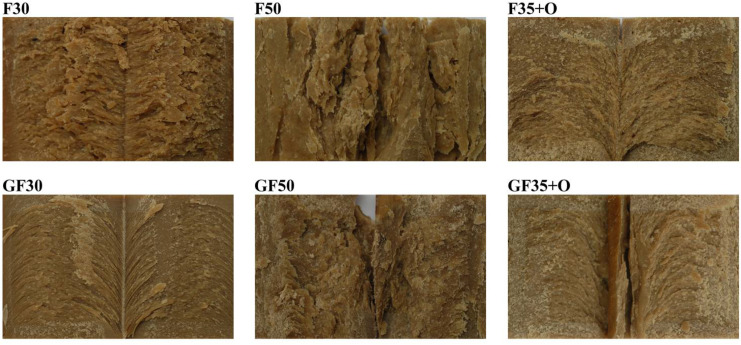
Photographs of the extrudates’ inner structure (see Table 1 for details of the samples).

Among extrudates containing faba bean groat, the extrudate containing 30% faba bean (F30) exhibited the highest hardness, gumminess, springiness, and chewiness, followed by extrudate containing oat fiber concentrate (F35+O) and extrudate containing 50% faba bean (F50) (Table 5). Extrudates with flour from germinated faba bean displayed a similar trend, with the highest values of hardness, gumminess, and chewiness observed in GF30, followed by GF35+O and GF50. However, there were no clear differences in springiness among these extrudates. Visual observations of the extrudate structures indicated that F30 and GF30 had the densest structure with more distinct fiber formation (Figure 2). Some layered structures were also observed in extrudates with OFC, while F50 and GF50 exhibited minimal noticeable fibers.

Furthermore, differences in mechanical properties were found when comparing extrudates with faba bean groat to extrudates with the same amount of flour from germinated faba bean (Table 5). At the lowest content of faba bean (30%), F30 was harder, gummier, and chewier than GF30. However, when the content of faba bean was higher (35 and 50%), the GF35 and GF50 samples were harder, gummier, and chewier.

The cutting strengths showed only minor differences between the extrudates (Table 5). When faba bean groat was used, F50 had the lowest longitudinal cutting strength, but no statistically significant differences were observed among extrudates with flour from germinated faba bean. Additionally, the use of germinated faba bean led to higher cutting strengths, although not all differences were statistically significant.

Regarding the water absorption capacity, the addition of OFC resulted in increased WAC (Table 5). When the content of faba bean groat was increased from 30 to 50%, WAC also increased, but the trend was the opposite when the content of flour from germinated faba bean was increased by the same amount.

### 3.4. Associations between the Sensory and Mechanical Properties

**Figure 3:**
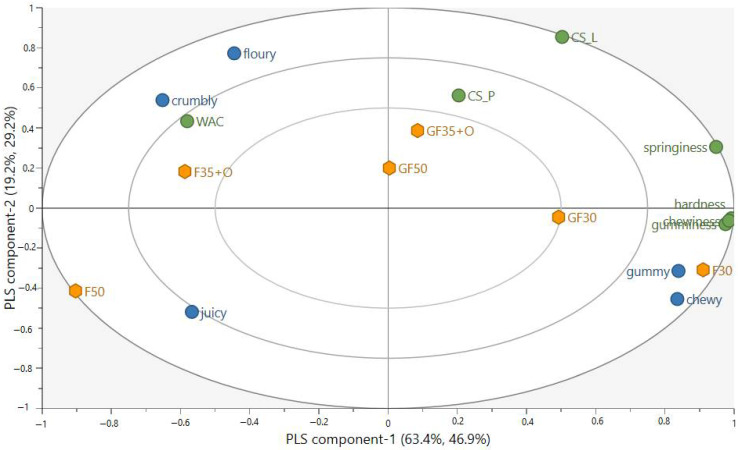
PLS bi-plot including extrudates (F30, F50, F35+O, GF30, GF50, and GF35+O), instrumental mechanical properties of hardness, gumminess, springiness, and chewiness, CS_L (longitudinal cutting strength), and CS_P (perpendicular cutting strength), WAC (water absorption capacity) and texture-related CATA attributes gummy, chewy, crumbly, and floury. PLS component-1 explains 62.7% of the variation in x and 53.9% of the variation in y, while PLS component-2 explains 19.5% of the variation in x and 27.4% of the variation in y.

Partial least squares regression (PLSR) analysis was applied to study the relationship between the texture-related CATA attributes and the mechanical properties and WAC of the extrudates (Figure 3). The PLSR included the first two components. The coefficient of determination (R^2^) for the model was 0.81, and the coefficient of prediction (Q^2^) was 0.45. The CATA attributes of ‘gummy’ and ‘chewy’ were correlated with each other and were associated with instrumental hardness, gumminess, springiness, and chewiness. In contrast, lower values of these mechanical properties were associated with ‘crumbly’ and ‘floury’ texture and higher water absorption capacity. The cutting strengths were not clearly associated with any of the CATA attributes.

## 4. Discussion

Our study was motivated by previous findings indicating that faba bean protein concentrate, a promising ingredient for plant-based meat analogs, is commonly perceived as intensely bitter, and that the bitter off-taste persists into the meat analogs made from the concentrate [13]. We investigated the possibility to avoid the bitterness problem by using commercially available faba bean groat/flour made from beans, which were either germinated or not, and heat-treated. We hypothesized that using these whole (i.e., not fractionated) flour/groat faba bean ingredients, together with PPI to increase the protein content to a sufficient level for proper structure formation in HME, we could produce meat analogs with an acceptable taste (without a bitter off-taste) and texture. Simultaneously, we were interested in the effects of the ratio of the ingredients (faba bean, PPI, and OFC) and the type of faba bean ingredient on the sensory and physical properties of the meat analogs.

Regarding taste, our hypothesis was supported by the results of the CATA profiling. ‘Bitter’ was, on average, the least frequently selected attribute, whereas ‘tasteless’ was cited rather often. ‘Umami’ and ‘meaty’ attributes could be beneficial for the acceptance of meat analogs, but the lack of these attributes may not be crucial, since it should be possible to add meaty and umami notes to products using flavorings. However, the extrudate samples were described as ‘beany’ by quite a few participants, implying that the beany flavor, typical of faba and other beans [33,34], needs to be addressed through other means, such as breeding [35], fermentation [36], or enzymes [37]. Nevertheless, the pleasantness of taste of the samples was rated, on average, around the middle of the scale (‘neither pleasant nor unpleasant’), suggesting that the samples did not present serious flavor challenges.

The raw materials and their ratios resulted in differences in the mechanical properties and water absorption capacity of the extrudates. The extrudates with the highest PPI content (F30 and GF30) had the highest hardness, gumminess, and chewiness, possibly due to the highest protein content creating a firm and dense structure [38]. Consistently among the results from the instrumental measurements, sample F30 was described most frequently as ‘gummy’ and ‘chewy’ by the sensory evaluation. Upon visual examination, these extrudates also showed the most distinct fiber formation. However, this did not seem to be associated with pleasantness of the mouthfeel, as the highest scores in this aspect were given to extrudate F50, which had the lowest values in the mechanical properties, less distinct fiber formation upon visual examination, and was frequently mentioned as ‘crumbly’, sometimes as ‘gummy’, but rarely as ‘chewy’. In contrast, the mouthfeel of the extrudates with highest PPI content was rated as less pleasant than that of the F50.

These results indicate that high hardness, chewiness, and gumminess are not desirable features in meat analogs, possibly resulting in less tender texture. Tenderness and juiciness are important attributes that increase consumer acceptance of meat products and are thus likely favorable features also in meat analogs as well [39].

In the current study, the addition of OFC increased the water absorption capacity of the extrudates, and simultaneously, they were described as ‘crumbly’ and ‘floury’ in the CATA profiling. The ability of dietary fiber such as oat *β*-glucan to absorb and retain water is well known. However, the addition of dietary fiber in food products can cause a floury mouthfeel and dryness [40,41]. Nevertheless, these effects are highly dependent on the amount and type of dietary fiber added.

Examination of the results on the differences in mechanical properties between extrudates containing the same amount of either faba bean groat (F extrudates) or flour from germinated faba bean (GF extrudates) showed some discrepancies. When the content of faba bean was 30%, extrudate F30 showed higher values of mechanical properties. However, when the content of faba bean increased, the GF extrudates were harder, gummier, and chewier. Due to the inconsistency of these results and the lack of specific information regarding the manufacturing process of the commercial raw materials used, no clear conclusions on the effect of germination of faba beans on the texture of the meat analogs could be drawn. However, germination can influence the technological properties of legumes such as pasting properties, water absorption capacity, emulsifying and foaming properties due to the hydrolysis of proteins and carbohydrates [42,43]. Furthermore, according to Guo et al. [19], using protein concentrate from germinated pea in high-moisture extrusion resulted in higher hardness. The same trend was observed in extrudates produced with protein concentrate from germinated lentil, although this difference was not statistically significant.

In terms of pleasantness, when the content of faba bean material was low (30%), no significant differences in any aspect of pleasantness were observed between the samples of different faba bean ingredient type (F30 and GF30). However, when the content of faba bean increased from 30% to 50%, pleasantness of mouthfeel and taste, and overall pleasantness increased at least nominally in the case of F extrudates, but decreased nominally in the case of GF extrudates. Consequently, F50 was rated as significantly more pleasant in mouthfeel and taste, and overall, than GF50.

We acknowledge that our study has certain limitations, which should be taken into consideration when interpreting the results. Our study employed only one batch of each ingredient, although it is known that many factors, such as variety, growth region, agronomic practices, seasonal variations, and post-harvest processes, can influence the exact composition of a specific batch [23,44,45]. Thus, future studies should expand upon our results using ingredients sourced from a wider range of faba bean, pea, and oat raw materials. Furthermore, replication of the extrudate preparation would have improved the study and allowed us to estimate test–retest reliability. However, that would have doubled the number of samples, and asking untrained panelists to evaluate large numbers of samples could lead to sensory fatigue and a decrease in motivation during the evaluation session. Our consumer sensory study involved a relatively low number of participants (55) and employed convenience sampling, both of which are suboptimal for consumer studies and undermine the generalizability of the findings. However, our aim was not to study acceptance of the extrudates in different consumer segments, but to focus on differences among the extrudates. With our design and methods, we were able to detect meaningful differences between the samples. Our samples in the sensory study were extrudates, which were served at room temperature without warming, seasoning, or other cooking. This may not represent the way that a consumer would eat meat analogs at home. However, even if the sensory evaluation of the samples in this manner may not represent a real-life eating situation, we chose this procedure to enable the participants to focus on the differences between the samples derived from the differences in their ingredients (and not mask the differences, or potential off-flavors, e.g., by seasoning). Finally, we acknowledge that it would have been useful to include control samples, such as a sample including faba bean flour from non-heat-treated beans, faba bean protein concentrate, or meat. However, the number of samples to be evaluated by untrained participants cannot be high to avoid sensory fatigue and subsequent biases. Future studies can complement our approach by employing a larger and more representative group of participants and serving meat analog samples with appropriate controls, cooked, and seasoned like a consumer would habitually eat them.

## 5. Conclusions

The bitter taste of meat analogs made from faba bean protein concentrate can be avoided by using faba bean groat/flour (together with PPI) instead of the concentrate. Meat analogs made from the faba bean groat/flour and PPI had mild flavor (often described as tasteless), enabling the addition of desirable flavors, such as umami, using added flavorings. However, beany flavor and lack of juiciness of these samples need to be addressed in future studies. Both sensory and instrumental texture analysis consistently showed that the samples with lower content of faba bean groat/flour (and respectively higher content of PPI and thus, protein) were gummier and chewier than the samples with higher faba bean content. A high faba bean content (50%) revealed differences in pleasantness between the samples made of different faba bean ingredient types, favoring the faba bean groat from non-germinated beans.

## Figures and Tables

**Table 1:** Composition and calculated protein content of the extrudate samples.

Sample Code	Content, % ^1^				Protein,
	F	GF	PPI	OFC	g/100 g DM ^2^
F30	30	0	70	0	62.2
GF30	0	30	70	0	62.9
F50	50	0	50	0	52.6
GF50	0	50	50	0	53.8
F35+O	35	0	35	30	43.7
GF35+O	0	35	35	30	44.6

^1^ F, faba bean (groat); GF, germinated faba bean (flour); PPI, pea protein isolate; OFC, oat fiber concentrate. ^2^ DM, dry matter. Calculated based on the measured protein content of each ingredient.

**Table 2:** Sensory attributes provided in the CATA task.

Sensory Modality	Attribute in English ^1^	Attribute in Finnish ^2^
Taste	Bitter	*Karvas*
	Sweet	*Makea*
	Tasteless	*Mauton*
	Umami	*Umami*
Flavor	Beany	*Papumainen*
	Cereal	*Viljainen*
	Meaty	*Lihaisa*
	Pea soup	*Hernekeitto*
	Toasted	*Paahtunut*
Texture (mouthfeel)	Chewy	*Sitkeä*
	Crumbly	*Mureneva*
	Gummy	*Kumimainen*
	Floury	*Jauhoinen*
	Juicy	*Mehukas*

^1^ Attributes are listed in alphabetical order within each sensory modality. ^2^ In our study, the attributes were provided to the participants in Finnish.

**Table 3:** Labels of the hedonic scale used to assess pleasantness of the samples.

Value in Data	Labels in English (Translation)	Labels in Finnish ^1^
1	Extremely unpleasant	*Erittäin epämiellyttävä*
2	Very unpleasant	*Hyvin epämiellyttävä*
3	Moderately unpleasant	*Melko epämiellyttävä*
4	Slightly unpleasant	*Hieman epämiellyttävä*
5	Neither pleasant nor unpleasant	*Ei miellyttävä eikä epämiellyttävä*
6	Slightly pleasant	*Hieman miellyttävä*
7	Moderately pleasant	*Melko miellyttävä*
8	Very pleasant	*Hyvin miellyttävä*
9	Extremely pleasant	*Erittäin miellyttävä*

^1^ In the score sheet of our study, the labels were provided in Finnish and arranged horizontally from left (1) to right (9) on the scale.

**Table 4:** Pleasantness of the extrudates.

Pleasantness ^1^		Sample						ANOVA		
		F30	GF30	F50	GF50	F35+O	GF35+O	F	df	*p*
Appearance	M	**5.2** ^c^	4.7 ^abc^	4.2 ^ab^	3.9 ^a^	5.0 ^bc^	4.7 ^abc^	4.06	5, 324	0.001
	(SD)	(1.8)	(1.8)	(1.9)	(1.6)	(1.7)	(1.8)			
Odor	M	4.9 ^a^	4.7 ^a^	4.3 ^a^	4.5 ^a^	**5.1** ^a^	5.0 ^a^	1.68	5, 324	0.140
	(SD)	(1.7)	(1.7)	(1.9)	(1.6)	(2.0)	(1.6)			
Texture (by hand)	M	3.7 ^a^	3.9 ^ab^	**4.8** ^b^	4.3 ^ab^	4.5 ^ab^	4.6 ^ab^	3.09	5, 323	0.010
	(SD)	(1.8)	(2.0)	(1.8)	(1.7)	(1.6)	(1.7)			
Mouthfeel	M	3.9 ^a^	4.6 ^a^	**5.8** ^b^	3.9 ^a^	4.3 ^a^	4.5 ^a^	6.66	5, 288	< 0.001
	(SD)	(2.0)	(2.1)	(1.9)	(1.9)	(2.0)	(1.7)			
Taste	M	4.7 ^ab^	4.6 ^ab^	**4.8** ^b^	3.8 ^a^	4.3 ^ab^	4.5 ^ab^	2.41	5, 288	0.037
	(SD)	(1.6)	(1.9)	(1.9)	(1.5)	(1.8)	(1.4)			
Overall	M	4.3 ^ab^	4.4 ^ab^	**5.1** ^b^	3.8 ^a^	4.4 ^ab^	4.6 ^ab^	3.88	5, 322	0.002
	(SD)	(1.8)	(1.7)	(1.7)	(1.5)	(1.6)	(1.5)			

^1^ Mean (SD) ratings by 55 participants (on a scale from 1 to 9). The nominally highest value in each aspect of pleasantness has been bolded. Means within the same row not sharing a common superscript letter are significantly different (Tukey’s test, *p* < 0.05).

**Table 5:** Mechanical properties and water absorption capacity of the extrudates.

Measure ^1^	F30	GF30	F50	GF50	F35+O	GF35+O
Hardness	375 ± 3 ^e^	340 ± 20 ^d^	250 ± 20 ^a^	306 ± 12 ^bc^	280 ± 20 ^ab^	310 ± 9 ^cd^
Gumminess	239 ± 14 ^e^	210 ± 9 ^d^	119 ± 14 ^a^	155 ± 9 ^b^	140 ± 13 ^ab^	182 ± 11^c^
Springiness	0.84 ± 0.03 ^d^	0.80 ± 0.03 ^cd^	0.664 ± 0.012 ^a^	0.785 ± 0.015 ^c^	0.73 ± 0.02 ^b^	0.80 ± 0.02 ^cd^
Chewiness	200 ± 20 ^e^	168 ± 12 ^d^	79 ± 9 ^a^	121 ± 5 ^b^	103 ± 11 ^b^	145 ± 8 ^c^
CS_L ^2^	9 ± 2 ^b^	10.4 ± 0.5 ^bc^	6.9 ± 0.9 ^a^	11.1 ± 1.5 ^bc^	9.6 ± 0.1 ^bc^	11.7 ± 0.8 ^c^
CS_P ^2^	6.6 ± 0.4 ^a^	11.1 ± 0.7 ^c^	7.2 ± 0.4 ^ab^	8 ± 2 ^abc^	8.0 ± 0.3 ^abc^	10.3 ± 0.6 ^c^
WAC ^3^	165 ± 2 ^a^	184 ± 6 ^abc^	192 ± 4 ^bc^	165.6 ± 1.5 ^ab^	220 ± 20 ^d^	208.3 ± 1.1 ^cd^

^1^ Means within the same row not sharing a common superscript letter are significantly different (Tukey’s test, *p* < 0.05). ^2^ CS_L, cutting strength, longitudinal; CS_P, cutting strength, perpendicular. ^3^ WAC, water absorption capacity.

## Data Availability

The original contributions presented in the study are included in the article/Supplementary Material (for the sensory data, see Table S1), further inquiries can be directed to the corresponding author.

## Supplementary Materials

The following supporting information can be downloaded at: https://www.mdpi.com/article/10.3390/foods13101444/s1, Table S1: Data from the sensory evaluation.
